# Independent histological validation of MR-derived radio-pathomic maps of tumor cell density using image-guided biopsies in human brain tumors

**DOI:** 10.1007/s11060-025-05105-x

**Published:** 2025-06-21

**Authors:** Gianluca Nocera, Francesco Sanvito, Jingwen Yao, Sonoko Oshima, Samuel A. Bobholz, Ashley Teraishi, Catalina Raymond, Kunal Patel, Richard G. Everson, Linda M. Liau, Jennifer Connelly, Antonella Castellano, Pietro Mortini, Noriko Salamon, Timothy F. Cloughesy, Peter S. LaViolette, Benjamin M. Ellingson

**Affiliations:** 1 UCLA Brain Tumor Imaging Laboratory (BTIL), Center for Computer Vision and Imaging Biomarkers, University of California Los Angeles, Los Angeles, CA USA; 2 Department of Radiological Sciences, David Geffen School of Medicine, University of California Los Angeles, Los Angeles, CA USA; 3 University Vita-Salute San Raffaele, Milan, Italy; 4 Neuroradiology Unit and CERMAC, IRCCS Ospedale San Raffaele, Milan, Italy; 5 Department of Neurosurgery and Gamma Knife Radiosurgery, IRCCS Ospedale San Raffaele, Milan, Italy; 6 Department of Diagnostic Imaging and Nuclear Medicine, Graduate School of Medicine, Kyoto University, Kyoto, Japan; 7 Department of Radiology, Medical College of Wisconsin, Milwaukee, WI USA; 8 Department of Neurosurgery, David Geffen School of Medicine, University of California, Los Angeles, CA USA; 9 Department of Neurology, Medical College of Wisconsin, Milwaukee, WI USA; 10 Department of Neurology, Ronald Reagan UCLA Medical Center, University of California, Los Angeles, CA USA; 11 David Geffen School of Medicine, UCLA Neuro-Oncology Program, University of California, Los Angeles, CA USA; 12 Department of Biomedical Engineering, Medical College of Wisconsin, Milwaukee, WI USA; 13 Department of Bioengineering, Henry Samueli School of Engineering and Applied Science, University of California Los Angeles, Los Angeles, CA USA; 14 Department of Psychiatry and Biobehavioral Sciences, David Geffen School of Medicine, University of California, Los Angeles, CA USA; 15 UCLA Radiology, UCLA Brain Tumor Imaging Laboratory, Los Angeles, USA

**Keywords:** Artificial intelligence, Diffusion imaging, Glioma, Histological validation, Imaging biomarker

## Abstract

**Purpose:**

In brain gliomas, non-invasive biomarkers reflecting tumor cellularity would be useful to guide supramarginal resections and to plan stereotactic biopsies. We aim to validate a previously-trained machine learning algorithm that generates cellularity prediction maps (CPM) from multiparametric MRI data to an independent, retrospective external cohort of gliomas undergoing image-guided biopsies, and to compare the performance of CPM and diffusion MRI apparent diffusion coefficient (ADC) in predicting cellularity.

**Methods:**

A cohort of patients with treatment-naïve or recurrent gliomas were prospectively studied. All patients underwent pre-surgical MRI according to the standardized brain tumor imaging protocol. The surgical sampling site was planned based on image-guided biopsy targets and tissue was stained with hematoxylin–eosin for cell density count. The correlation between MRI-derived CPM values and histological cellularity, and between ADC and histological cellularity, was evaluated both assuming independent observations and accounting for non-independent observations.

**Results:**

Sixty-six samples from twenty-seven patients were collected. Thirteen patients had treatment-naïve tumors and fourteen had recurrent lesions. CPM value accurately predicted histological cellularity in treatment-naïve patients (b = 1.4, R^2^ = 0.2, *p* = 0.009, rho = 0.41, *p* = 0.016, RMSE = 1503 cell/mm^2^), but not in the recurrent sub-cohort. Similarly, ADC values showed a significant association with histological cellularity only in treatment-naive patients (b = 1.3, R^2^ = 0.22, *p* = 0.007; rho = -0.37, *p* = 0.03), not statistically different from the CPM correlation. These findings were confirmed with statistical tests accounting for non-independent observations.

**Conclusion:**

MRI-derived machine learning generated cellularity prediction maps (CPM) enabled a non-invasive evaluation of tumor cellularity in treatment-naïve glioma patients, although CPM did not clearly outperform ADC alone in this cohort.

**Supplementary Information:**

The online version contains supplementary material available at 10.1007/s11060-025-05105-x.

## Introduction

High-grade gliomas, the most common primary brain malignancy [1], are primarily treated through surgical resection of the contrast-enhancing tumor, when feasible [2]. While many studies showed that a radical surgical resection of the contrast-enhancing region warrants a survival benefit [3–7], recent findings advocate for a “supramarginal” resections involving the surrounding peri-enhancing tissue, as tumor cells are known to infiltrate beyond visible enhancement and tumor often recurs in the peri-cavitary region [8–10]. When surgery is not feasible due to tumor location or multifocality, stereotactic biopsy is essential for accurate diagnosis and treatment planning [2, 11]. In both scenarios, non-invasive biomarkers of tumor cellularity could prove valuable, guiding the extent of resection in operable cases and identifying biopsy targets in inoperable ones.

Apparent Diffusion Coefficient maps (ADC) derived from diffusion-weighted imaging (DWI) is commonly considered as a non-invasive surrogate biomarker for tumor cellularity [4, 12–19], showing a moderate/strong inverse correlation with histopathological cellularity in recent meta-analyses (pooled Spearman’s correlation coefficient around –0.66) [20, 21]. ADC was proven to potentially improve the identification of areas of high cellularity in the peri-enhancing tissue [13, 22, 23]. Nevertheless, there is significant variability of findings across studies, with some authors reporting poor or even opposite relationships [24–26], possibly due to ADC values being also influenced by confounding microstructural factors other than tumor cellularity in gliomas [27].

Machine learning algorithms could represent a new approach for non-invasive tumoral cellularity prediction. Recently, a random forest ensemble algorithm was developed, that generates voxel-wise radio-pathomic maps reflecting tissue cellularity [28]. This model was trained with multiparametric conventional and diffusion MRI obtained pre-mortem to predict the histological cell count from co-registered autopsy samples from brain slices aligned with imaging. In validation studies, radio-pathomic maps could distinguish hypercellular areas within the contrast-enhancing region, identify regions of increased cellularity in the peri-enhancing tumor regions [23, 28], and provide information on who may benefit from bevacizumab [29]. Most recently, these models were applied to large publicly available imaging cohorts to explore the relationship between perfusion metrics and cell density [30].

In the current study, we aim to validate this previously-developed machine learning algorithm on an external independent cohort whose images and histopathological samples were obtained at a separate institution. In more detail, multiparametric MRI datasets served as input for the algorithm generating cellularity prediction maps (CPM), and the CPM values from image-guided biopsy targets were compared to histopathological cellularity. Moreover, since ADC is an established imaging biomarker for tumor cellularity, we compared the performance of the CPM with that of the ADC map in predicting histological cell density.

## Methods

### Patient selection

Patients gave written informed consent to participate in a prospective IRB-approved imaging study (IRB#14–001261). All patients had either a radiological recurrence of a previously-diagnosed glioma or a treatment-naïve radiologically suspected glioma. Pre-surgical MRI scans for these patients were acquired between April 2015 and November 2018 at the Department of Radiology of the University of California Los Angeles. All patients underwent open craniotomy for surgical resection. The precise sampling site was planned based on image-guided biopsy targets and stored for further analysis. The inclusion criteria for the current study were as follows: histopathological diagnosis of adult-type diffuse glioma, availability of pre-surgical MRI scans, and availability of digitalized hematoxylin and eosin staining (H&E).

### Magnetic resonance imaging acquisition and pre-processing

All patients underwent a pre-surgical acquisition on a 3 T Siemens Prisma (Siemens Healthineers) scanner according to the standardized brain tumor imaging protocol (BTIP) [31, 32], including parameter-matched pre- and post-contrast 3D T_1_-weighted images with 1-mm isotropic voxels (T1 and T1CE), 2D T_2_-weighted FLAIR images with 3-mm slice thickness (FLAIR), diffusion tensor imaging (DTI) with 2-mm isotropic voxels (64 direction, *b*-value = 1000 s/mm^2^). ADC was calculated on the scanner from DTI data. A single dose of Gadavist (Gadobutrol, Bayer) (~ 0.1 mL/kg) was administered at a rate of ~ 4 mL/s during the acquisition of T_2_*-weighted dynamic susceptibility images (DSC), in compliance with guidelines [33], as per BTIP [31]. Relative cerebral blood volume (rCBV) maps were calculated using a bidirectional leakage correction algorithm, as previously described [34]. DSC was not part of the machine learning algorithm.

T1, T1CE, FLAIR, and ADC images were the input sequences for the machine learning algorithm generating cellularity prediction maps (CPM), previously trained and validated on MRI and autopsy glioblastoma data [23, 28], and applied using a custom Matlab script (MathWorks). The voxel-wise CPM values output by the algorithm were divided by the whole-brain intensity SD at the single patient level, to normalize values across patients [28]. Each image-guided biopsy target consisted in a single spherical region-of-interest (ROI) with diameter 5 mm. Mean normalized CPM values and mean ADC values were extracted from each ROI.

### Histopathological analysis

Samples were hematoxylin and eosin stained (H&E) and digitalized. All recurrent specimens exhibited “active” tumor cells with mitoses and ki-67 positivity, therefore no cases of pure radiation necrosis were included. QuPath (https://qupath.github.io, v05.1), an open-source software application for digital pathology, was used for semi-automatic cell counting [35]. Each stain was manually revised, and cell count parameters were adjusted to supervise the cell count. All cells identified on the H&E stain were counted and included in the cellularity quantitation, independently from their ki-67 expression.

### Statistical analysis

*Group comparisons.* The Mann-Withey test was used to analyze group differences in CPM values (e.g., samples from contrast-enhancing tumor vs samples from non-enhancing tumor).

*Cellularity correlations, assuming independent observations.* The linear relationship (Pearson’s correlation) and rank correlation (Spearman’s correlation) between CPM cellularity (CPM cell/mm^2^) and histological cellularity in H&E stain (H&E cell/mm^2^) were analyzed assuming that all biopsy-derived measurements were independent. For Pearson’s correlations, observations considered as outliers were detected using ROUT method [36] and were excluded from the analysis. The performance of CPM prediction was evaluated using root mean square error (RMSE) values. Since ADC is commonly considered a proxy of tumor cellularity [24, 37], the correlations between ADC and histological cellularity were also tested. The correlation between CPM and histological cellularity was directly compared to the correlation between ADC and histological cellularity, using Meng’s z-tests. These analyses under the assumption of independent observations were conducted as a first-line approach that is easier to visualize and interpret.

*Cellularity correlations, accounting for non-independent observations.* Since multiple measurements were obtained from the same patient (and from the same MRI scan), the assumption of independent observations is inherently violated. To account for non-independent observations (e.g., two biopsy targets obtained from the same patient), two additional analyses were performed. First, a mixed-effects model was built to test the linear relationship between histological cellularity and CPM values (fixed effect) while accounting for the patient ID (random effect). Second, an iterative approach was performed by randomly picking a single sample per patient on each iteration, and repeated for 1000 iterations to ultimately obtain a distribution of the Spearman’s coefficients (rho) that did not include clustered observations. These analyses accounting for non-independent observations were conducted to confirm the results of the first analysis which assumed independent observations.

## Results

### Patients’ cohort characteristics

Twenty-seven patients met the inclusion criteria; thirteen were treatment-naïve (TN), and fourteen recurrent (R) tumors; sixty-six tumor tissue samples were collected from this cohort. Thirty-seven samples were obtained from enhancing tissue and twenty-nine from the non-enhancing tumor. Table 1 summarizes the clinical, pathological, and radiological characteristics of the enrolled cohort.

**Table 1:** Demographic, clinical, and radiological characteristics

Patient characteristics	Value
Number of patients	27
Sex n (%)	M 17 (63%) F 10 (37%)
Age, (mean ± SD)	49.4 ± 15.6
Prior treatments	
Treatment-naïve (%)	13 (48%)
Recurrent (%)	14 (52%)
Histology	
WHO CNS4, 2016 (n, %)	Oligoastrocytoma Grade II, 1 (4%) Astrocytoma Grade II, 4 (15%) Anaplastic Oligoastrocytoma Grade III, 5 (18%) Anaplastic Oligodendroglioma Grade III, 1 (4%) Anaplastic Astrocytoma Grade III, 1 (4%) Glioblastoma Grade IV, 14 (51%) High-grade glioma Grade IV, 1 (4%)
WHO CNS5, 2021 (n, %)	Astrocytoma Grade 2, 4 (15%) Astrocytoma Grade 3, 4 (15%) Oligodendroglioma Grade 3, 1 (3%) Astrocytoma Grade 4, 5 (19%) Glioblastoma Grade. 4, 11 (41%) NOS*, 2 (7%)

Samples characteristics	Value
Number of samples	66
Location (n, %)	Frontal 27 (41%) Parietal 4 (6%) Fronto-parietal 6 (9%) Temporal 13 (20%) Occipital 5 (8%) Fronto-temporo-insular 9 (14%) Multifocal 2 (3%)
Number of samples per patient	1 sample (7 patients) 2 samples (6 patients) 3 samples (9 patients) 4 samples (5 patients)
Radiologic appearance of the sample	
Enhancing (n, %)	37 (56%)
Non enhancing (n, %)	29 (44%)
H&E cellularity (cell/mm^2^, mean ± sd)	2856 ± 1461
CPM cellularity (cell/mm^2^, mean ± sd)	1323 ± 324

*In two patients it was not possible to presume the WHO CNS5 2021 classification, due to insufficient information about their molecular status

### Descriptive statistics and group comparisons

**Fig. 1:**
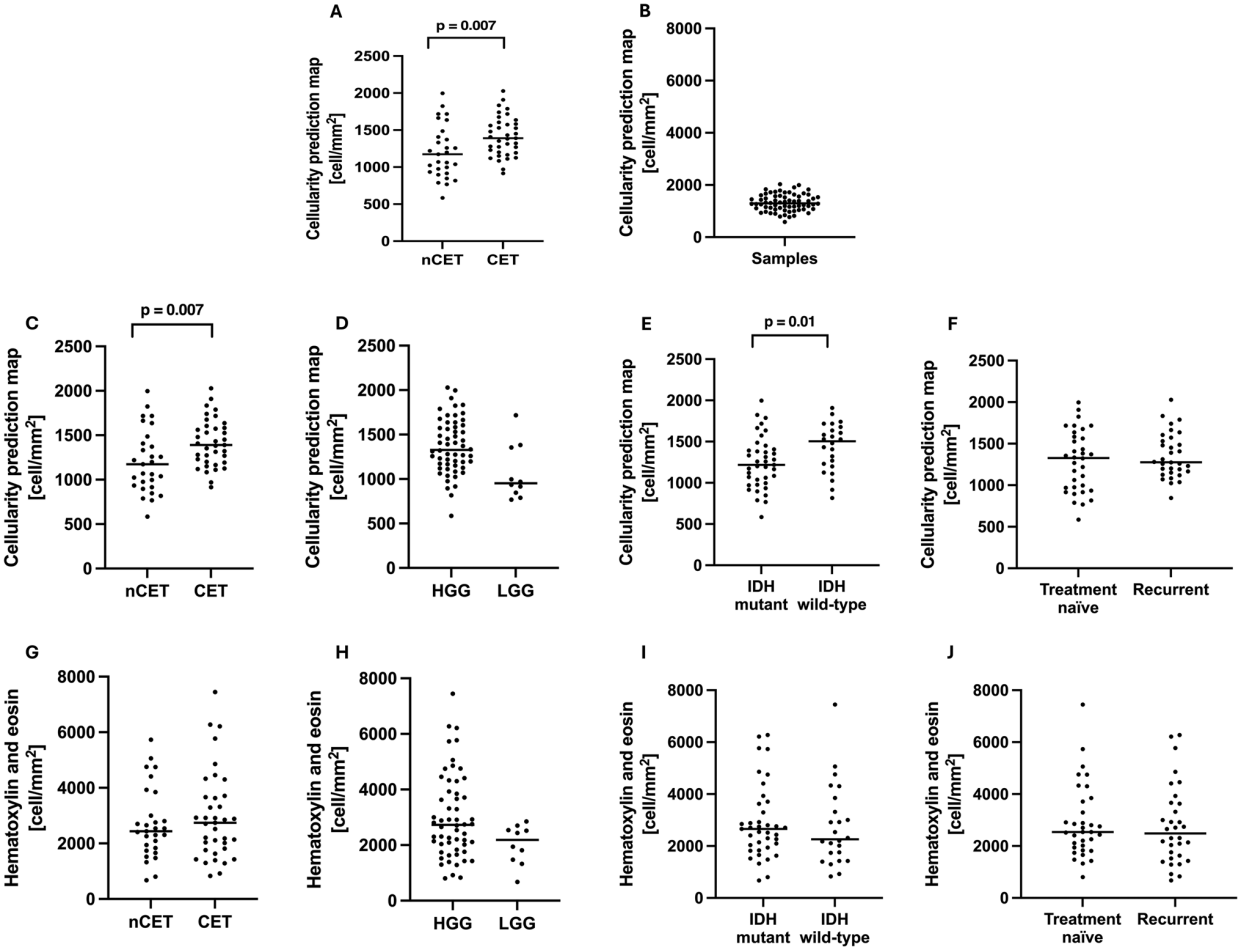
Descriptive statistics of sample cellularity and group differences. **A** Histological cell density using H&E staining (H&E cellularity). **B** Cellularity predicted by the radio-pathomic maps (CPM cellularity). **C**–**G** Difference in CPM values (**C**) and H&E cellularity (**G**) between samples taken from enhancing (CET) vs non-enhancing (nCET) tumor components. **D**–**H** Difference in CPM values (**D**) and H&E cellularity (**H**) between samples taken from high- vs low-grade gliomas (HGG vs LGG). **E**–**I** Difference in CPM values (**E**) and H&E cellularity (**I**) between samples taken from IDH mutant and IDH wild-type gliomas. **F**–**J** Differences in CPM values (**F**) and H&E cellularity (**J**) between samples taken from treatment-naïve and recurrent lesions

The mean H&E cellularity from histology was 2856 cell/mm^2^ (SD 1461 cell/mm^2^) across all surgical samples (Fig. 1a). The mean predicted cellularity value from the MRI-derived CPM map was 1323 cell/mm^2^ (SD 324.4 cell/mm^2^) (Fig. 1b). Samples taken from CET regions showed significantly higher CPM values compared to the ones taken from nCET (mean: 1415 cell/mm^2^ vs 1206 cell/mm^2^ *p* = 0.007, Fig. 1c). CPM values tended to be higher in HGG than in LGG (mean: 1358 cell/mm^2^ vs 1195 cell/mm^2^, Fig. 1d), although this difference was not significant. Samples from IDH wild-type tumors had significantly higher CPM values compared to those from IDH mutant tumors (mean: 1432 cell/mm^2^ vs 1243 cell/mm^2^, *p* = 0.01, Fig. 1e). Finally, CPM values corresponding to samples from treatment-naïve lesions and from recurrent lesions did not exhibit a statistical difference (mean 1290 cell/mm^2^ vs 1358 cell/mm^2^, Fig. 1f).

### Cellularity correlations assuming independent observations

**Fig. 2:**
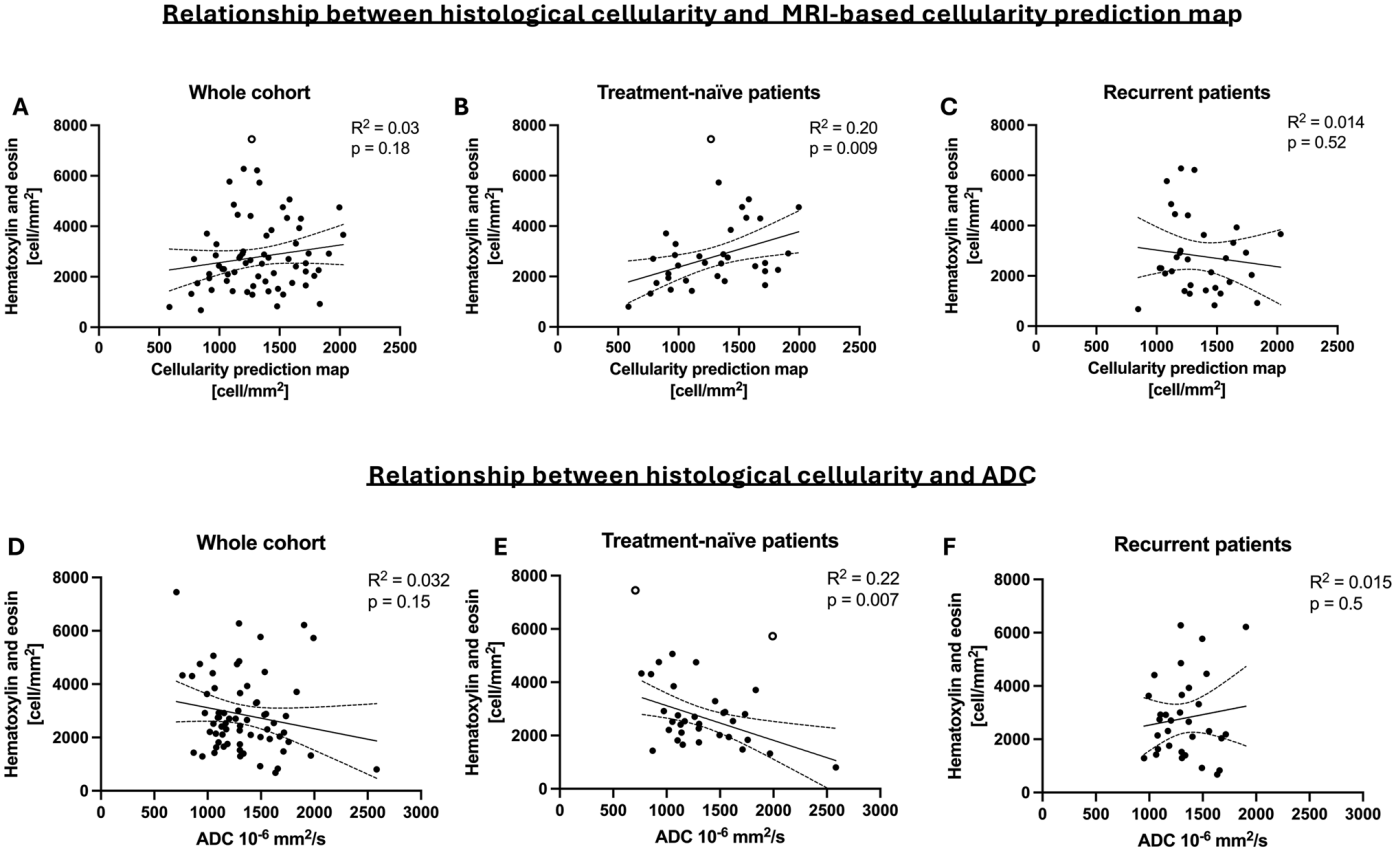
Relationship between CPM cellularity from radio-pathomic maps, ADC values, and histological H&E cellularity. The radio-pathomic map performs well in predicting the histological cellularity of treatment-naïve tumors (**B**), while no association was found in recurrent patients (**C**) and in the whole cohort (**A**). Similarly, ADC was associated with histological cellularity only in the treatment-naïve cohort (**E**), and not in the whole cohort (**D**) nor the recurrent subset (**F**). White dots represent outliers

CPM was a predictor of H&E cellularity when considering the subset of treatment-naïve lesions (TN), showing both a significant linear correlation (b = 1.4, R^2^ = 0.2, *p* = 0.009, RMSE = 1503 cell/mm^2^) and rank correlation (rho = 0.41, *p* = 0.016) (Table 2, Fig. 2b). Instead, no significant association was found between CPM values and H&E cellularity in the whole cohort (b = 0.7, R^2^ = 0.028, *p* > 0.05, RMSE 1974 cell/mm^2^; rho = 0.17, *p* > 0.05, Fig. 2a), and considering only patients with recurrent disease (b = -0.66, R^2^ = 0.014, *p* > 0.05, RMSE 3156 cell/mm^2^; rho = -0.12, *p* > 0.05, Fig. 2c).

**Table 2:** Comparison between radio-pathomic map and ADC statistics

	Metrics	Value (*p*)		
		Whole cohort	Treatment-naïve	Recurrent
CPM	b (Pearson’s)	0.7 (> 0.05)	1.4 (0.009)*	− 0.66 (> 0.05)
	R^2^ (Pearson’s)	0.028	0.20	0.014
	rho (Spearman’s)	0.17 (> 0.05)	0.41 (0.016)*	− 0.12 (> 0.05)
	RMSE (cell/mm^2^)	1974	1503	3156
ADC	b (Pearson’s)	− 0.78 (> 0.05)	− 1.3 (0.007)*	0.78 (> 0.05)
	R^2^ (Pearson’s)	0.032	0.22	0.031
	rho (Spearman’s)	− 0.18 (> 0.05)	− 0.37 (0.03)*	− 0.18 (> 0.05)

*b* regression coefficient, *R*^2^ coefficient of determination, *Rho* Spearman’s correlation coefficient, *RSME* root mean square error, *ns* not significant

Similarly, the ADC map showed a significant association with H&E cellularity in treatment-naïve patients (b = 1.3, R^2^ = 0.22, *p* = 0.007; rho = -0.37, *p* = 0.03, Fig. 2e), while no association was found in the whole cohort (b = -0.78, R^2^ = 0.032, *p* = 0.15; rho = -0.18, *p* > 0.05, Fig. 2d) and in recurrent lesions (b = 0.78, R^2^ = 0.031, *p* > 0.05; rho = -0.18, *p* > 0.05, Fig. 2f).

The correlation between CPM and histological cellularity was not statistically different from the correlation between ADC and histological cellularity (z = 0.27, *p* = 0.79 in the treatment-naïve subgroup).

### Cellularity correlations accounting for non-independent observations

Additional analyses accounting for non-independent observations confirmed the results of the independent sample approach. With the linear mixed model (Supplemental Table 1), CPM values were statistically-significant predictors of H&E cellularity only in the treatment-naïve cohort (b = 1.41, *p* = 0.013), and not significant predictors in the whole cohort and in recurrent cases. Similarly, the association between ADC values and H&E cellularity was significant only in treatment-naïve patients (b = -1.21, *p* = 0.01).

The results of the iterative approach (Supplemental Fig. 1) revealed a higher correlation between CPM and H&E cellularity in treatment-naïve patients (mean rho = 0.51) than in the entire cohort (mean rho = 0.25) or in the recurrent group (mean rho = 0.22). Similarly, the association between ADC values and H&E cellularity was stronger in treatment-naïve patients (mean rho = -0.37) compared to the entire cohort (mean rho = -0.22) and to the recurrent group (mean rho = -0.08).

### Representative cases

**Fig. 3:**
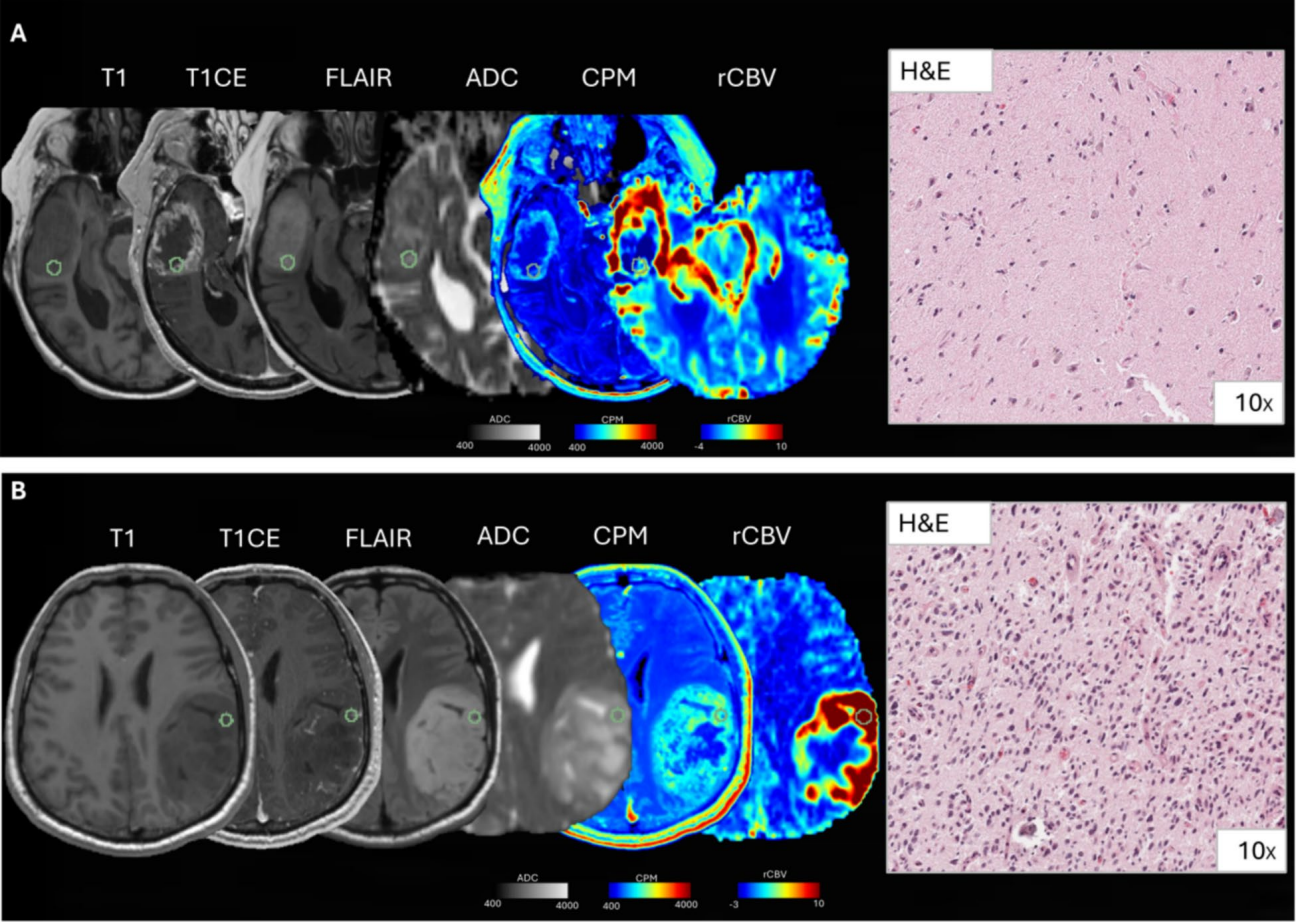
Representative cases of good agreement between radio-pathomic maps and histology. **A** Sample collected from the enhancing tumor component. CPM values were low, and this finding was associated with low histological cellularity at H&E stain. Interestingly rCBV values were not elevated. **B** Sample taken from non-enhancing tumoral tissue. CPM values are elevated, and a high histological cellularity is found on H&E. rCBV values were increased in this region

A 86-year-old female received a first diagnosis of right temporal lobe glioblastoma (IDH wild-type, grade 4, Fig. 3a). In the targeted surgical region, obtained from contrast-enhancing tumor tissue, CPM maps exhibited relatively low values of predicted cellular density (916 cells/mm^2^), and ADC was 1145 × 10^–6^ mm^2^/s. Histological analysis revealed a relatively low tumor cellularity (2112 cells/mm^2^). A 28-year-old male had a treatment-naïve non-enhancing astrocytoma in the left fronto-parietal area (IDH mutated, 1p19q intact, grade 3, Fig. 3b). In the targeted region, CPM maps exhibited high values of predicted cellular density (1983 cells/mm^2^), and ADC was 1127 × 10^–6^ mm^2^/s. Histological analysis revealed a relatively high tumor cellularity (4749 cells/mm^2^). These two cases show successful prediction of H&E histology using CPM maps.

**Fig. 4:**
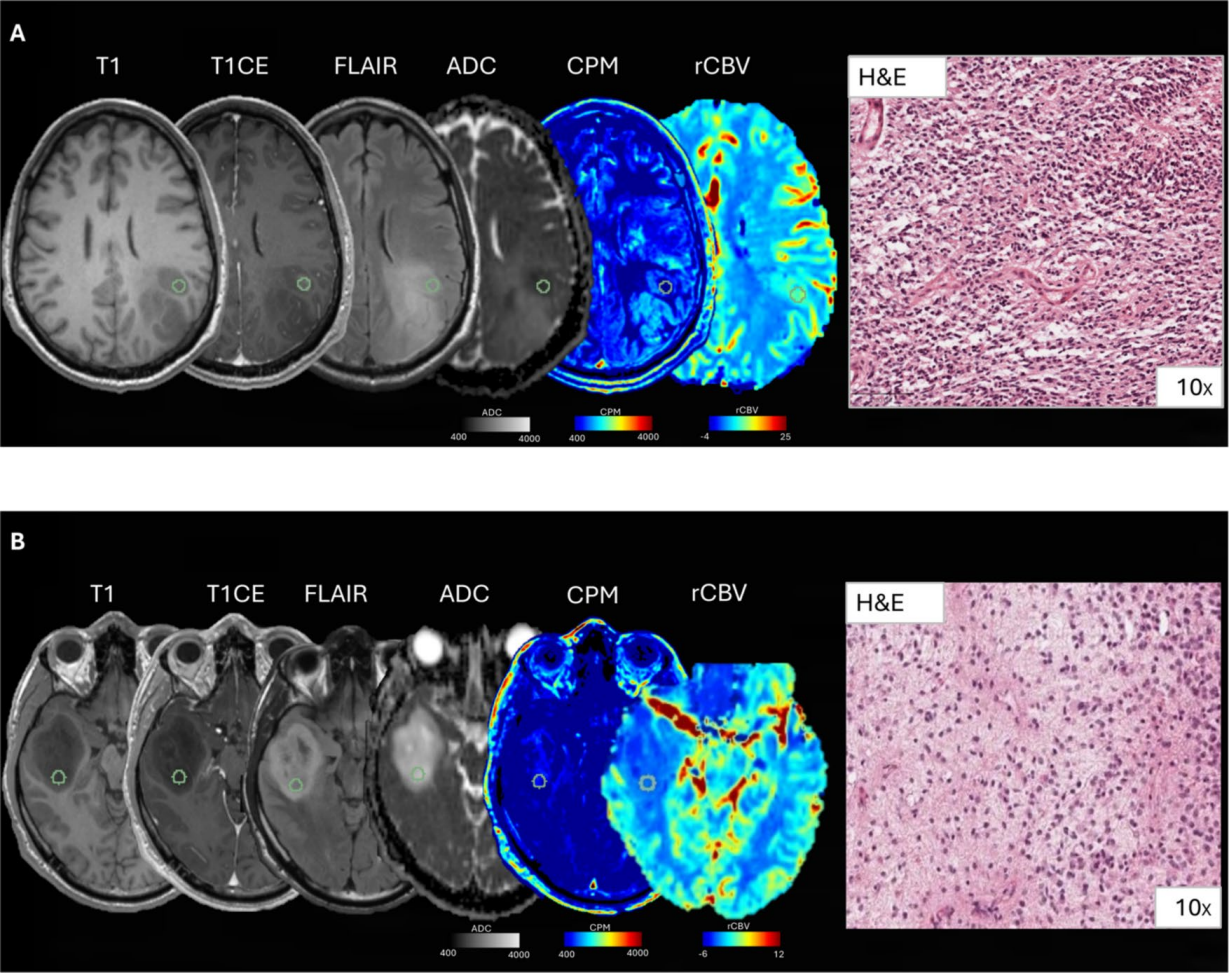
Representative cases of poor agreement between radio-pathomic maps and histology. **A** Sample collected from non-enhancing tumoral tissue. In this case, the CPM map fails to predict the high cellularity found in histological analysis. **B** Sample collected from non-enhancing tumoral tissue. The CPM map shows low values of predicted cellularity, while H&E showed a moderate to high histological cellularity. In both cases, rCBV values were not elevated

A 35-year-old female presented with a treatment-naïve left frontal glioblastoma (IDH wild-type, grade 4, Fig. 4a). In the targeted surgical sample, obtained non-enhancing tumor tissue, CPM maps exhibited moderate values of predicted cellular density (1240 cells/mm^2^) and ADC was 685 × 10^–6^ mm^2^/s. However, histological analysis revealed extremely high tumor cellularity (7449 cells/mm^2^). A 24-year-old female received a first diagnosis of right fronto-temporo-insular astrocytoma (IDH mutated, 1p19q intact, grade 4, Fig. 4b), non-enhancing. In the targeted region, CPM maps exhibited moderate values of predicted cellular density (1354 cells/mm^2^) and ADC was 1992 × 10^–6^ mm^2^/s. However, histological samples revealed a high tumor cellularity (5732 cells/mm^2^). In these latter two cases CPM map fail to accurately predict histological cellularity.

## Discussion

Results from this study suggest that CPM values provide valuable information about tumor cellularity in treatment-naïve patients, while the recurrent sub-cohort showed poor association with histological findings. Similarly, ADC values showed an association with histological tumor cellularity in the treatment-naïve sub-cohort (with a slightly weaker correlation compared to CPM), but not in the recurrent sub-cohort.

In treatment-naïve gliomas, identifying highly cellular areas, particularly beyond the contrast-enhancing margin, could help to depict the areas of tumoral infiltration, guiding surgical resection and optimizing adjuvant treatment, as radiotherapy [2]. Maximal safe resection improves patients’ prognosis [2], and recent evidence demonstrates that the additional removal of infiltrating tumor components in the peri-enhancing region (“supramarginal resection”), whenever feasible, leads to a better prognosis in high-grade gliomas [38–41]. Since peri-enhancing T2-weighted/FLAIR abnormalities alone do not directly reflect tumor cellularity [38, 41, 42], imaging biomarkers depicting areas of increased cellularity in the peri-enhancing region would enable surgeons to remove infiltrated tumor tissue while minimizing the risk of postoperative neurological deficits related to the increased extent of resection. Interestingly, 34% of samples in our study obtained from high-grade gliomas were collected from non-enhancing areas. The RMSE calculated only in this subset of samples (1777 cells/mm^2^) was lower than the ones of the whole cohort (1974 cell/mm^2^) indicating a smaller error of the model in the prediction of the histopathologic cell density in this specific group (Supplemental Fig. 2). This finding is particularly noteworthy, suggesting that CPM could serve as a valuable biomarker for predicting tumor cellularity beyond the contrast-enhancing regions, potentially guiding “supramarginal” resections. In addition, non-invasive biomarkers reflecting tumor cellularity would also be useful in non-resectable treatment-naïve tumors, as they would improve the planning of stereotactic biopsies, which should target the most aggressive areas for a more accurate histopathological diagnosis.

ADC from DWI is commonly used as a non-invasive biomarker for tumor cellularity, but its correlation with histological cellularity is highly variable in the literature [20, 21, 24–26, 43]. This could be due to microstructural factors other than cell density, that influence ADC values, such as extracellular matrix composition, vasogenic edema, degenerative changes, and tissue compression [27, 44, 45]. CPM demonstrated a positive moderate correlation with H&E cellularity in treatment-naïve patients, possibly outperforming ADC when accounting for non-independent observations.

In the recurrent setting, too, the non-invasive evaluation of tumor cellularity is clinically relevant. After adjuvant chemoradiation, distinguishing between *true* tumor progression and treatment-induced effects (also referred to as “pseudoprogression”, and which can include radiation necrosis) is challenging [46]. Current solutions include advanced imaging such as perfusion-weighted imaging and amino-acid PET, and the use of confirmatory scans as suggested in the Response Assessment in Neuro-Oncology (RANO) criteria 2.0 [47, 48]. However, confirmation scans delay the diagnosis of tumor progression [49], and the routine use of advanced imaging is limited to a few centers and suffers from technical limitations that limit their universalizability [50]. Since *true* tumor progression is marked by actively mitotic tumor cells, linked to an increase in local cellularity [51–54], imaging surrogates of cellularity would arguably aid the distinction between tumor progression and pseudoprogression. Some meta-analyses reported encouraging results supporting the role of diffusion imaging for this application [55, 56], but a pooled cut-off ADC value obtained from multiple studies (1330 × 10^–6^ mm^2^/s) showed a poor diagnostic performance overall, due to an overlap of ADC values between groups [56]. CPM maps could represent a valid alternative to evaluate tumor cellularity and identify areas of true tumor progression when monitoring treated gliomas. However, CPM performed worse in recurrent patients, and the RMSE value in this subset (3156 cell/mm^2^) was remarkably higher than in the treatment-naïve subset (1503 cell/mm^2^), indicating a greater error of the model in the prediction of histopathological cellularity in this specific subgroup. This increased error, as well as a non-significant correlation, shows that CPM maps may not be reliable predictors of histopathological cellularity in the recurrent setting. This finding can appear counterintuitive, since the CPM machine learning algorithm was trained on datasets collected mostly from recurrent tumor [28]. However, the scenario of chemoradiation-treated recurrent glioblastomas is complex from a radiographic standpoint, since actively-growing tumor tissue is often mixed with areas of treatment-related effects, presenting overlapping MRI features. Additionally, our patients with recurrent lesions received different therapies, which potentially induced heterogenous radiographic changes. Overall, these factors related to prior treatments may explain why the current CPM model may not reliably predict cellularity in this patient subset. Notably, in line with prior meta-analyses [55, 56], ADC was not a significant predictor of tumor cellularity in the recurrent sub-cohort in our study, either. Our findings, as well as data from the literature, suggest that *currently* MRI-based prediction of tumor cellularity in the recurrent setting with either ADC or CPM may be unsatisfactory. Integrating multi-parametric imaging, such as perfusion-weighted imaging, metabolic imaging, or advanced diffusion models, into new machine learning algorithms predicting tumor cellularity may potentially help overcoming this limitation, by providing more pathophysiological information related to active tumor growth and treatment effects.

### Limitations

The relatively small sample size of our cohort could have reduced the power of the statistical analyses. The diverse histological characteristics within this cohort may have introduced additional variability based on the different tumor subtypes. The samples were collected during surgery using pre-operative imaging. However, some inherent limitations in the precision of the correspondence between pre-operatory imaging and sampling site were inevitable, due to the changes in the operative field related to brain shift and surgical manipulation.

## Conclusion

In this study, we tested a previously-developed machine learning algorithm for MRI-based cellularity prediction on an external independent cohort and compared its performance with that of the ADC maps. Machine learning generated cellularity prediction maps (CPM) were valuable for tumor cellular prediction in treatment-naïve patients in samples collected from both contrast-enhancing and non-enhancing areas. In recurrent patients, the association between CPM and histological cellularity was poor. Our results suggest that machine learning algorithms exploiting the information from multi-parametric MRI data have the potential to enhance tumor cellularity prediction in gliomas, with potential applications in guiding stereotactic biopsy sampling and identifying tumor-infiltrated non-enhancing areas for tailored supramarginal surgical resections. However, the performance of CPM maps did not show clear superiority over the ADC maps for the prediction of histological cellularity in this cohort.

## Supplementary Information

Below is the link to the electronic supplementary material.

Supplementary file1 (PDF 1309 KB)

## Data Availability

Data from this cohort is available from the authors upon request.

## References

[1] Ostrom QT et al (2023) CBTRUS statistical report: primary brain and other central nervous system tumors diagnosed in the United States in 2016–2020. Neuro Oncol 25(12 Suppl 2):iv1–iv99 37793125 10.1093/neuonc/noad149 PMC10550277

[2] Weller M et al (2021) EANO guidelines on the diagnosis and treatment of diffuse gliomas of adulthood. Nat Rev Clin Oncol 18(3):170–186 33293629 10.1038/s41571-020-00447-z PMC7904519

[3] Blakstad H et al (2023) Survival in a consecutive series of 467 glioblastoma patients: Association with prognostic factors and treatment at recurrence at two independent institutions. PLoS ONE 18(2):e0281166 36730349 10.1371/journal.pone.0281166 PMC9894455

[4] Ellingson BM et al (2010) Validation of functional diffusion maps (fDMs) as a biomarker for human glioma cellularity. J Magn Reson Imaging 31(3):538–548 20187195 10.1002/jmri.22068 PMC2903058

[5] Hertler C et al (2023) Long-term survival with IDH wildtype glioblastoma: first results from the ETERNITY Brain Tumor Funders’ Collaborative Consortium (EORTC 1419). Eur J Cancer 189:112913 37277265 10.1016/j.ejca.2023.05.002

[6] Mineo JF et al (2007) Prognosis factors of survival time in patients with glioblastoma multiforme: a multivariate analysis of 340 patients. Acta Neurochir (Wien) 149(3):245–252 (**discussion 252-3**) 17273889 10.1007/s00701-006-1092-y

[7] Simpson JR et al (1993) Influence of location and extent of surgical resection on survival of patients with glioblastoma multiforme: results of three consecutive radiation therapy oncology group (RTOG) clinical trials. Int J Radiat Oncol Biol Phys 26(2):239–244 8387988 10.1016/0360-3016(93)90203-8

[8] Certo F et al (2021) FLAIRectomy in supramarginal resection of glioblastoma correlates with clinical outcome and survival analysis: a prospective, single institution. Case Ser Oper Neurosurg (Hagerstown) 20(2):151–163 10.1093/ons/opaa293 33035343

[9] Karschnia P et al (2023) Prognostic validation of a new classification system for extent of resection in glioblastoma: a report of the RANO resect group. Neuro Oncol 25(5):940–954 35961053 10.1093/neuonc/noac193 PMC10158281

[10] Hou LC et al (2006) Recurrent glioblastoma multiforme: a review of natural history and management options. Neurosurg Focus 20(4):E5 16709036 10.3171/foc.2006.20.4.2

[11] Louis DN et al (2021) The 2021 WHO Classification of Tumors of the Central Nervous System: a summary. Neuro Oncol 23(8):1231–1251 34185076 10.1093/neuonc/noab106 PMC8328013

[12] Romano A et al (2023) Diffusion weighted imaging in neuro-oncology: diagnosis, post-treatment changes, and advanced sequences-an updated review. Cancers (Basel) 15(3):618 36765575 10.3390/cancers15030618 PMC9913305

[13] Durand-Muñoz C et al (2019) Pre-operative apparent diffusion coefficient values and tumour region volumes as prognostic biomarkers in glioblastoma: correlation and progression-free survival analyses. Insights Imaging 10(1):36 30887267 10.1186/s13244-019-0724-8 PMC6423260

[14] Sugahara T et al (1999) Usefulness of diffusion-weighted MRI with echo-planar technique in the evaluation of cellularity in gliomas. J Magn Reson Imaging 9(1):53–60 10030650 10.1002/(sici)1522-2586(199901)9:1<53::aid-jmri7>3.0.co;2-2

[15] Karavaeva E et al (2015) Relationship between [18F]FDOPA PET uptake, apparent diffusion coefficient (ADC), and proliferation rate in recurrent malignant gliomas. Mol Imaging Biol 17(3):434–442 25465392 10.1007/s11307-014-0807-3

[16] Chenevert TL et al (2000) Diffusion magnetic resonance imaging: an early surrogate marker of therapeutic efficacy in brain tumors. J Natl Cancer Inst 92(24):2029–2036 11121466 10.1093/jnci/92.24.2029

[17] Gupta RK et al (2000) Relationships between choline magnetic resonance spectroscopy, apparent diffusion coefficient and quantitative histopathology in human glioma. J Neurooncol 50(3):215–226 11263501 10.1023/a:1006431120031

[18] Hayashida Y et al (2006) Diffusion-weighted imaging of metastatic brain tumors: comparison with histologic type and tumor cellularity. AJNR Am J Neuroradiol 27(7):1419–1425 16908550 PMC7977525

[19] Lyng H, Haraldseth O, Rofstad EK (2000) Measurement of cell density and necrotic fraction in human melanoma xenografts by diffusion weighted magnetic resonance imaging. Magn Reson Med 43(6):828–836 10861877 10.1002/1522-2594(200006)43:6<828::aid-mrm8>3.0.co;2-p

[20] Chen L et al (2013) The correlation between apparent diffusion coefficient and tumor cellularity in patients: a meta-analysis. PLoS ONE 8(11):e79008 24244402 10.1371/journal.pone.0079008 PMC3823989

[21] Surov A, Meyer HJ, Wienke A (2017) Correlation between apparent diffusion coefficient (ADC) and cellularity is different in several tumors: a meta-analysis. Oncotarget 8(35):59492–59499 28938652 10.18632/oncotarget.17752 PMC5601748

[22] Wu J et al (2022) Analysis of DWI in the classification of glioma pathology and its therapeutic application in clinical surgery: a case-control study. Transl Cancer Res 11(4):805–812 35571647 10.21037/tcr-22-114 PMC9091004

[23] Bobholz SA et al (2024) Noninvasive autopsy-validated tumor probability maps identify glioma invasion beyond contrast enhancement. Neurosurgery 95(3):537–547 38501824 10.1227/neu.0000000000002898 PMC11302944

[24] Eidel O et al (2016) Automatic analysis of cellularity in glioblastoma and correlation with ADC using trajectory analysis and automatic nuclei counting. PLoS ONE 11(7):e0160250 27467557 10.1371/journal.pone.0160250 PMC4965093

[25] Jenkinson MD et al (2010) Cellularity and apparent diffusion coefficient in oligodendroglial tumours characterized by genotype. J Neurooncol 96(3):385–392 19618117 10.1007/s11060-009-9970-9

[26] Stadlbauer A et al (2006) Gliomas: histopathologic evaluation of changes in directionality and magnitude of water diffusion at diffusion-tensor MR imaging. Radiology 240(3):803–810 16926329 10.1148/radiol.2403050937

[27] Rose S et al (2013) Correlation of MRI-derived apparent diffusion coefficients in newly diagnosed gliomas with [18F]-fluoro-L-dopa PET: what are we really measuring with minimum ADC? AJNR Am J Neuroradiol 34(4):758–764 23079407 10.3174/ajnr.A3315 PMC7964500

[28] Bobholz SA et al (2022) Radio-pathomic maps of cell density identify brain tumor invasion beyond traditional MRI-defined margins. AJNR Am J Neuroradiol 43(5):682–688 35422419 10.3174/ajnr.A7477 PMC9089258

[29] Bobholz SA et al (2024) Radio-pathomic maps of glioblastoma identify phenotypes of non-enhancing tumor infiltration associated with bevacizumab treatment response. J Neurooncol 167(2):233–241 38372901 10.1007/s11060-024-04593-7 PMC11024025

[30] Bobholz SA et al (2025) Multi-site retrospective analysis of diffusion and perfusion mri correlates to glioma characteristics derived from radio-pathomic maps. Neuro Oncol. 10.1093/neuonc/noaf044 39960860 10.1093/neuonc/noaf044 PMC12417833

[31] Sanvito F et al (2023) Standardized brain tumor imaging protocols for clinical trials: current recommendations and tips for integration. Front Radiol 3:1267615 38152383 10.3389/fradi.2023.1267615 PMC10751345

[32] Ellingson BM et al (2015) Consensus recommendations for a standardized brain tumor imaging protocol in clinical trials. Neuro Oncol 17(9):1188–1198 26250565 10.1093/neuonc/nov095 PMC4588759

[33] Welker K et al (2015) ASFNR recommendations for clinical performance of MR dynamic susceptibility contrast perfusion imaging of the brain. AJNR Am J Neuroradiol 36(6):E41-51 25907520 10.3174/ajnr.A4341 PMC5074767

[34] Leu K et al (2016) Bidirectional contrast agent leakage correction of dynamic susceptibility contrast (DSC)-MRI improves cerebral blood volume estimation and survival prediction in recurrent glioblastoma treated with bevacizumab. J Magn Reson Imaging 44(5):1229–1237 26971534 10.1002/jmri.25227

[35] Bankhead P et al (2017) QuPath: open source software for digital pathology image analysis. Sci Rep 7(1):16878 29203879 10.1038/s41598-017-17204-5 PMC5715110

[36] Motulsky HJ, Brown RE (2006) Detecting outliers when fitting data with nonlinear regression—a new method based on robust nonlinear regression and the false discovery rate. BMC Bioinform 7:123 10.1186/1471-2105-7-123 PMC1472692 16526949

[37] Ellingson BM et al (2018) Validation of postoperative residual contrast-enhancing tumor volume as an independent prognostic factor for overall survival in newly diagnosed glioblastoma. Neuro Oncol 20(9):1240–1250 29660006 10.1093/neuonc/noy053 PMC6071654

[38] Li YM et al (2016) The influence of maximum safe resection of glioblastoma on survival in 1229 patients: can we do better than gross-total resection? J Neurosurg 124(4):977–988 26495941 10.3171/2015.5.JNS142087

[39] de Leeuw CN, Vogelbaum MA (2019) Supratotal resection in glioma: a systematic review. Neuro Oncol 21(2):179–188 30321384 10.1093/neuonc/noy166 PMC6374756

[40] Molinaro AM et al (2020) Association of maximal extent of resection of contrast-enhanced and non-contrast-enhanced tumor with survival within molecular subgroups of patients with newly diagnosed glioblastoma. JAMA Oncol 6(4):495–503 32027343 10.1001/jamaoncol.2019.6143 PMC7042822

[41] Pessina F et al (2017) Maximize surgical resection beyond contrast-enhancing boundaries in newly diagnosed glioblastoma multiforme: is it useful and safe? A single institution retrospective experience. J Neurooncol 135(1):129–139 28689368 10.1007/s11060-017-2559-9

[42] Castellano A et al (2021) Advanced imaging techniques for radiotherapy planning of gliomas. Cancers (Basel) 13(5):1063 33802292 10.3390/cancers13051063 PMC7959155

[43] Higano S et al (2006) Malignant astrocytic tumors: clinical importance of apparent diffusion coefficient in prediction of grade and prognosis. Radiology 241(3):839–846 17032910 10.1148/radiol.2413051276

[44] Patel KS et al (2020) Decorin expression is associated with predictive diffusion MR phenotypes of anti-VEGF efficacy in glioblastoma. Sci Rep 10(1):14819 32908231 10.1038/s41598-020-71799-w PMC7481206

[45] Pope WB et al (2012) Differential gene expression in glioblastoma defined by ADC histogram analysis: relationship to extracellular matrix molecules and survival. AJNR Am J Neuroradiol 33(6):1059–1064 22268080 10.3174/ajnr.A2917 PMC8013245

[46] Ellingson BM et al (2017) Pseudoprogression, radionecrosis, inflammation or true tumor progression? Challenges associated with glioblastoma response assessment in an evolving therapeutic landscape. J Neurooncol 134(3):495–504 28382534 10.1007/s11060-017-2375-2 PMC7893814

[47] Wen PY et al (2023) RANO 2.0: update to the response assessment in neuro-oncology criteria for high- and low-grade gliomas in adults. J Clin Oncol 41(33):5187–5199 37774317 10.1200/JCO.23.01059 PMC10860967

[48] Sanvito F et al (2024) RANO 2.0 criteria: concepts applicable to the neuroradiologist’s clinical practice. Curr Opin Oncol 36(6):536–544 39011735 10.1097/CCO.0000000000001077 PMC11493521

[49] van den Elshout R et al (2023) Apparent diffusion coefficient metrics to differentiate between treatment-related abnormalities and tumor progression in post-treatment glioblastoma patients: a retrospective study. Cancers (Basel) 15(20):4990 37894355 10.3390/cancers15204990 PMC10605800

[50] Patel P et al (2017) MR perfusion-weighted imaging in the evaluation of high-grade gliomas after treatment: a systematic review and meta-analysis. Neuro Oncol 19(1):118–127 27502247 10.1093/neuonc/now148 PMC5193025

[51] Li C et al (2023) Clinical significance of histopathological features of paired recurrent gliomas: a cohort study from a single cancer center. BMC Cancer 23(1):8 36597096 10.1186/s12885-022-10484-9 PMC9811748

[52] Kim JH et al (2012) Pathologic diagnosis of recurrent glioblastoma: morphologic, immunohistochemical, and molecular analysis of 20 paired cases. Am J Surg Pathol 36(4):620–628 22441548 10.1097/PAS.0b013e318246040c

[53] Haider AS et al (2020) Toward a standard pathological and molecular characterization of recurrent glioma in adults: a response assessment in neuro-oncology effort. Neuro Oncol 22(4):450–456 31844891 10.1093/neuonc/noz233 PMC7158649

[54] Woodworth GF et al (2013) Histopathological correlates with survival in reoperated glioblastomas. J Neurooncol 113(3):485–493 23666202 10.1007/s11060-013-1141-3 PMC3994532

[55] Tsakiris C et al (2020) Differentiation between true tumor progression of glioblastoma and pseudoprogression using diffusion-weighted imaging and perfusion-weighted imaging: systematic review and meta-analysis. World Neurosurg 144:e100–e109 32777397 10.1016/j.wneu.2020.07.218

[56] van den Elshout R et al (2022) Diffusion imaging could aid to differentiate between glioma progression and treatment-related abnormalities: a meta-analysis. Insights Imaging 13(1):158 36194373 10.1186/s13244-022-01295-4 PMC9532499

