# Supplementary material for: Independent histological validation of MR-derived radio-pathomic maps of tumor cell density using image-guided biopsies in human brain tumors
Source: J Neurooncol. 2025 Jun 21;175(1):111–22. doi: 10.1007/s11060-025-05105-x (PMC12367939; doi:10.1007/s11060-025-05105-x)
Supplement: Supplementary file 1 — Supplementary file1 (PDF 1309 KB) [file 11060_2025_5105_MOESM1_ESM.pdf]

Independent histological validation of MR-derived radio-pathomic maps of tumor cell density using image-guided biopsies in human brain tumors

Journal of Neuro-Oncology

Supplemental Table 1. Statistical analysis accounting for non-independent observations. An iterative approach has been used to extract 1000 different subsets of samples formed by a single sample from each patient. Then, the distribution of Spearman correlation coefficients between H&E cellularity and CPM cellularity and ADC values was calculated (upper side of the table). In addition, a mixed-effects model (lower side of the table) was built to evaluate the linear relationships between CPM cellularity and H&E cellularity, and between ADC and H&E cellularity, while accounting for the random effect due to multiple intra-patient observations. TN= treatment-naïve. SEM = standard error of the mean.

	**Rho coefficients from iterative approach**					
	**Whole cohort**		**Treatment-naïve**		**Recurrent**	
	**Mean**	**SEM**	**Mean**	**SEM**	**Mean**	**SEM**
**CPM**	0.25	0.0034	0.51	0.0043	0.022	0.0046
**ADC**	-0.22	0.0047	-0.37	0.0047	-0.08	0.0062
	**Beta coefficients from the mixed-effects model**					
	**Whole cohort**		**Treatment-naïve**		**Recurrent**	
	**Coefficient**	**p-value**	**Coefficient**	**p-value**	**Coefficient**	**p-value**
**CPM**	0.78	0.14	1.41	0.013*	-0.4	0.7
**ADC**	-0.51	0.3	-1.21	0.01*	1.07	0.35

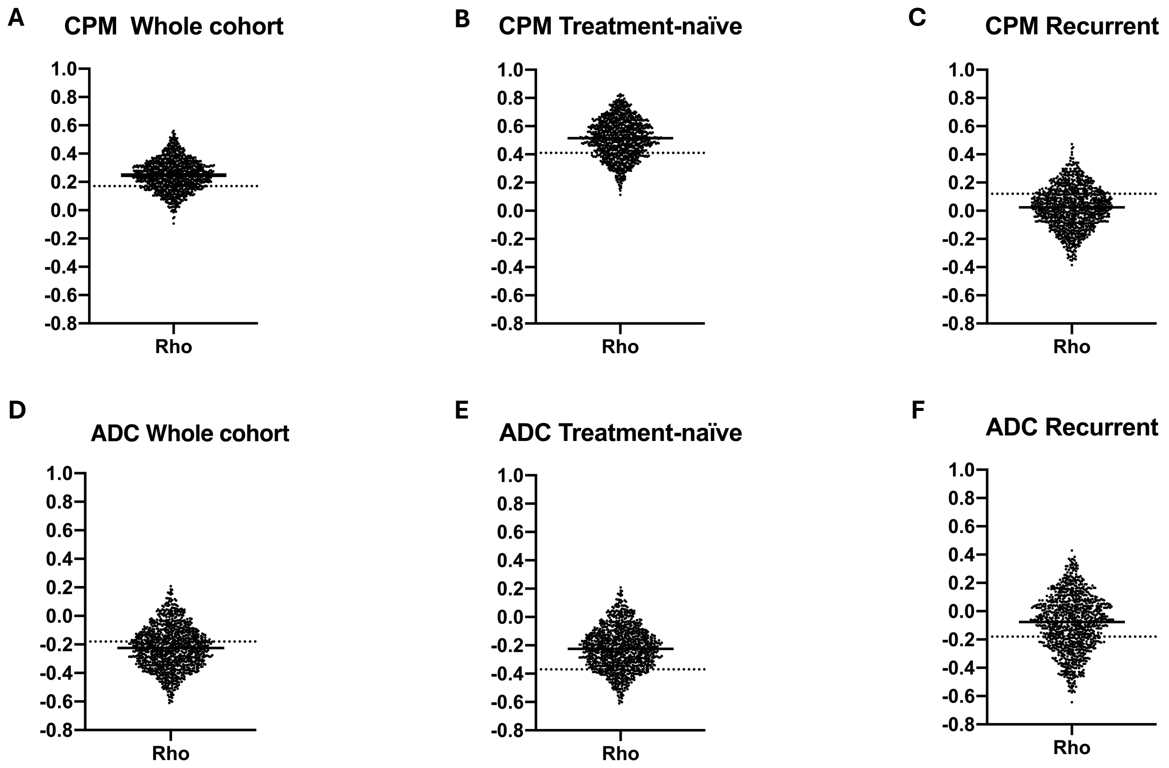

Supplemental Figure 1. Distribution of the Spearman correlation coefficient found with the iterative approach between cellularity prediction maps (CPM) and histological cellularity and between ADC and histological cellularity

Relationship between histological cellularity and MRI-based cellularity prediction map

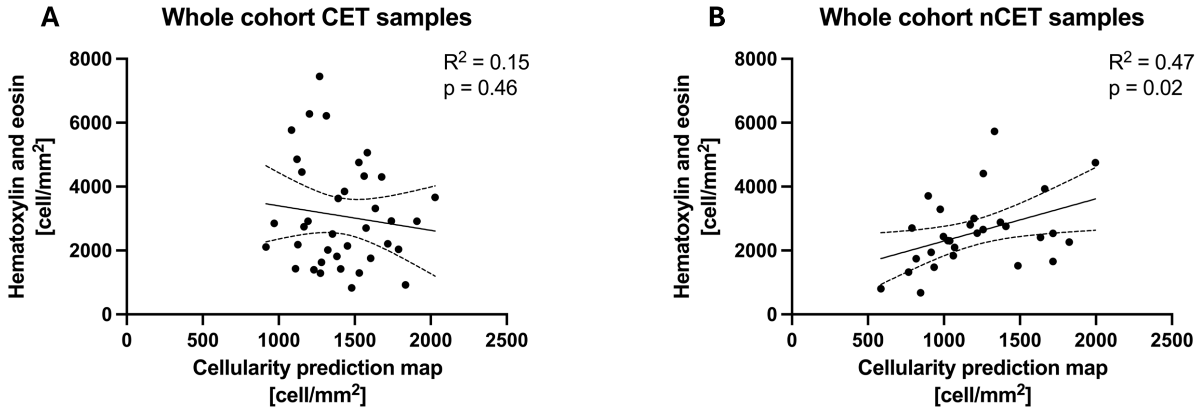

Supplemental Figure 2. Relationship between CPM cellularity from radio-pathomic maps, and histological H&E cellularity in samples stratified by the radiological appearance of sampling location. The radio-pathomic map performs better in predicting the histological cellularity of samples taken from the non-enhancing component of the tumor (B), compared to the enhancing region (A).
